# Research progress on coronary heart disease and cytokines: mechanisms, clinical implications, and future directions

**DOI:** 10.1186/s13019-026-04332-1

**Published:** 2026-06-04

**Authors:** Liang Zhang, Jinpeng Xu, Guangcheng Sun

**Affiliations:** Department of Cardiology, Anhui Provincial Chest Hospital, No. 397 Jixi Road, Hefei, 230011 China

**Keywords:** Coronary heart disease, Cytokines, Mechanisms, Clinical implications, Prognosis

## Abstract

Coronary heart disease (CHD) is a prevalent cardiovascular condition with increasing prevalence and mortality, where inflammation plays a key pathogenic role. Cytokines, critical mediators of immune and inflammatory responses, are closely involved in CHD progression by regulating inflammation, myocardial apoptosis, endothelial dysfunction, and vascular smooth muscle cell proliferation. It introduces cytokines’ basic properties, elaborates on pro-inflammatory (TNF-α, IL-1, IL-6) and anti-inflammatory (IL-10, TGF-β) cytokines’ specific effects on atherosclerosis and cardiomyocyte function, and discusses their clinical value in risk assessment, prognosis, and targeted therapy. Existing research gaps and future directions (e.g., advanced technologies, personalized therapies) are highlighted. This review provides a comprehensive overview to inform CHD basic research and clinical management.

## Introduction

The prevalence and mortality associated with CHD have been on the rise, prompting extensive research into its underlying mechanisms and potential therapeutic targets. Some signaling molecules are involved in inflammatory responses, apoptosis of myocardial cells, endothelial dysfunction, and proliferation of vascular smooth muscle cells, all of which contribute to the progression of CHD. Understanding the intricate interplay between cytokines and these pathological processes can unveil novel therapeutic strategies aimed at mitigating the impact of this disease [1].

Cytokines such as interleukins, tumor necrosis factor-α (TNF-α), and chemokines have been identified as key players in the inflammatory processes that exacerbate atherosclerosis. For instance, interleukin-1 β (IL-1β) has been shown to promote inflammation within atherosclerotic plaques, while TNF-α is associated with endothelial dysfunction and increased vascular permeability [1]. The CANTOS trial demonstrated that targeting IL-1β with monoclonal antibodies significantly reduced cardiovascular events in patients with stable coronary artery disease, underscoring the potential of cytokine-targeted therapies in managing CHD [1]. Moreover, the identification of biomarkers related to cytokine levels presents opportunities for early diagnosis and risk stratification in patients susceptible to CHD, facilitating timely interventions and improved outcomes [2].

**Fig. 1:**
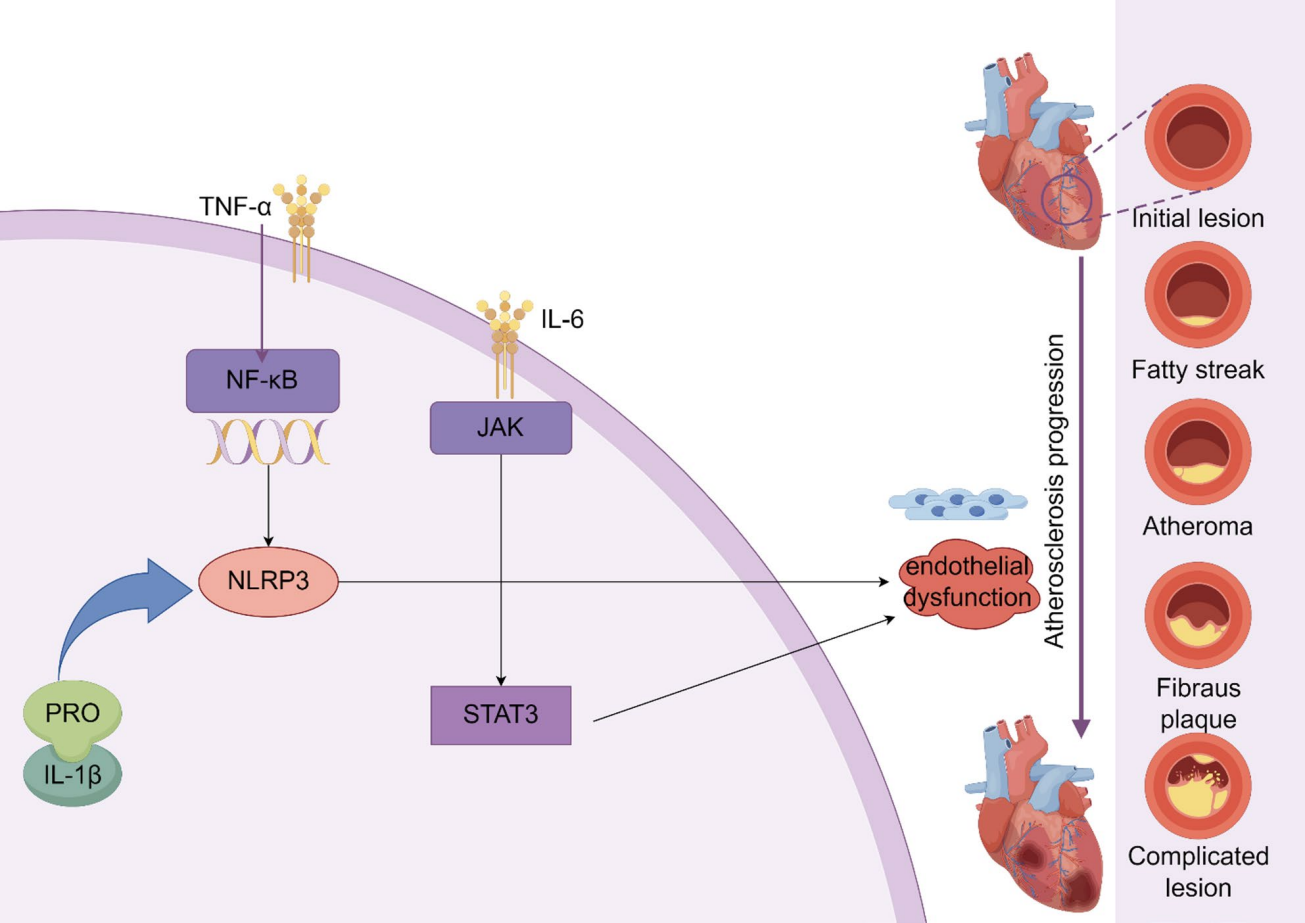
The Role of Cytokines in the Progression of Coronary Heart Disease

The future direction of research in CHD focuses on elucidating the specific pathways through which cytokines exert their effects, particularly their roles in modulating immune responses and influencing cellular behavior within the cardiovascular system (Fig. 1). For example, the NLRP3 inflammasome has emerged as a critical regulator of inflammatory responses in atherosclerosis, with potential therapeutic implications for its inhibition [3]. Additionally, the exploration of traditional Chinese medicine (TCM) and its active compounds offer promising avenues for developing novel treatments that target cytokine signaling pathways, aiming to reduce inflammation and improve vascular health [4].

In conclusion, cytokines play a pivotal role in the pathogenesis of coronary heart disease through their involvement in inflammatory responses and vascular dysfunction (Table 1). By leveraging advancements in molecular biology and pharmacology, healthcare providers may be able to offer more effective and personalized treatment options, ultimately reducing the burden of this prevalent cardiovascular condition [5]. Although there is currently a lot of research on the relationship between coronary heart disease and cytokines, the mechanism of cytokines and coronary heart disease is not fully understood. These unresolved issues highlight the urgency of exploring the complex regulatory network of cytokines and developing high-sensitivity biomarkers, which is also the core direction of this review.

**Table 1:** The relationship between various cytokines and coronary heart disease

Research area	Key cytokines /Markers/Technology	Core findings/Advances	Significance/Future direction
**Pathogenesis**	Pro-inflammatory: TNF-α, IL-1β, IL-6	Promote arterial wall inflammation, endothelial dysfunction, smooth muscle proliferation, exacerbating atherosclerosis.	Understand the core mechanisms of disease development.
	Anti-inflammatory: IL-10, TGF-β	Inhibit inflammatory responses, promote plaque stability, exerting protective effects.	Identify endogenous protective mechanisms and intervention targets.
**Biomarkers**	Classic: IL-6, CRP	Levels significantly correlate with CHD severity and risk of adverse cardiovascular events.	Important tools for assessing disease risk and prognosis.
	Novel: GDF-15	Show potential in CHD prognostic assessment.	Develop more precise prognostic markers.
**Targeted Therapy**	Validated: IL-1β (targeted antibody)	CANTOS trial: Targeting IL-1β significantly reduced cardiovascular event risk in stable CHD patients.	Confirm clinical benefits of anti-inflammatory therapy.
	In Development: TNF-α, IL-6 inhibitors (e.g., JAK)	Novel therapeutics (e.g., JAK inhibitors) are under development.	Expand scope of targeted therapy, explore more effective drugs.

### Basic concepts and classification of cytokines

#### Definition of cytokines

Cytokines are a diverse group of soluble proteins that play crucial roles in cell signaling within the immune system. They are produced by a variety of cells, including immune cells, endothelial cells, and fibroblasts, and they function as mediators of immune responses. Cytokines can be classified into several categories based on their functions and the types of cells they influence. They include interleukins, interferons, tumor necrosis factors, growth factors, and chemokines. Each cytokine has specific roles in the regulation of immune responses, inflammation, and hematopoiesis. The complex interplay between different cytokines is essential for maintaining homeostasis in the immune system, as well as for the initiation and resolution of inflammatory processes [6, 7].

#### Major types of cytokines

Cytokines can be broadly categorized into pro-inflammatory and anti-inflammatory cytokines. Pro-inflammatory cytokines, such TNF-α, IL-1, and IL-6, are primarily involved in promoting inflammation and mobilizing immune responses against pathogens. Conversely, anti-inflammatory cytokines, such as IL-10 and transforming growth factor-β (TGF-β), help to resolve inflammation and promote tissue repair. Other important categories include chemokines, which are involved in the recruitment of immune cells to sites of inflammation, and growth factors, which regulate cell proliferation and differentiation. The balance between these various cytokines is crucial for the proper functioning of the immune system and the prevention of diseases such as autoimmune disorders and chronic inflammatory conditions [8, 9].

#### Functions and mechanisms of action of cytokines

Cytokines exert their effects through specific receptors on target cells, triggering a cascade of intracellular signaling pathways that lead to various biological responses. These responses can include the activation of immune cells, the promotion of cell proliferation, and the induction of apoptosis. For instance, IL-6 can stimulate the differentiation of B cells into antibody-producing plasma cells, while TNF-α can induce apoptosis in certain cell types. Furthermore, cytokines can influence the expression of other cytokines and modulate the activity of immune cells, creating a feedback loop that amplifies or dampens immune responses. Dysregulation of cytokine signaling can lead to pathological conditions, such as cytokine storms, which are characterized by excessive inflammatory responses that can result in tissue damage and multi-organ failure [10, 11]. Understanding the intricate roles of cytokines in health and disease is essential for developing targeted therapies for various immune-mediated disorders [10, 12]. The role of inflammation mediated by NLRP3 in atherosclerosis was summarized. The expression level of NLRP3 was positively correlated with atherosclerosis risk factors and major adverse cardiac events. Moreover, NLRP3 is an independent predictor of major adverse cardiac events, and it has good predictive value for major adverse cardiac events [13].

### The role of cytokines in coronary heart disease

#### Pro-inflammatory cytokines and their relationship with coronary atherosclerosis

Pro-inflammatory cytokines play a pivotal role in the pathogenesis of coronary atherosclerosis, which is a key contributor to CHD. Elevated levels of cytokines such as TNF-α, IL-1, and IL-6 have been associated with increased inflammation within the arterial walls, leading to the progression of atherosclerotic lesions. Studies have shown that these cytokines promote the expression of adhesion molecules on endothelial cells, facilitating the recruitment of inflammatory cells, particularly monocytes and macrophages, to the site of injury. This inflammatory cascade not only exacerbates the atherosclerotic process but also contributes to plaque instability, which can result in acute coronary events such as myocardial infarction [14]. Furthermore, the presence of adipokines, such as resistin, has been linked to the upregulation of pro-inflammatory cytokines, thereby aggravating atherosclerosis [15]. Mitsis et al. summarized relevant studies that showed that the increased levels of adipokines adiponectin, visfatin and resistin were related to the high mortality and incidence rate of myocardial infarction [16]. The interplay between these cytokines and various risk factors, including dyslipidemia and hypertension, underscores the complex mechanisms driving the development of coronary artery disease. Cytokines have been proven to have a promoting effect on plaque formation in coronary heart disease. Different types of cytokines participate in the whole process leading to the instability of atherosclerotic plaque, which leads to the occurrence of myocardial infarction. Especially IL-1 and TNF-α families are involved in the accumulation of inflammatory cells, formation of vulnerable plaques, platelet aggregation, myocardial cell apoptosis, and adverse remodeling after myocardial infarction [17]. IL-1, including IL-1a and IL-1b, promotes the occurrence of inflammatory reactions [18]. A meta-analysis suggests that the TNF-α G308A variant may be associated with an increased risk of coronary heart disease [19]. A prospective study suggests that multiple cytokines are associated with the risk of coronary heart disease, showing an approximately logarithmic linear relationship [18].

#### The protective role of anti-inflammatory cytokines

While pro-inflammatory cytokines contribute to the pathogenesis of coronary heart disease, anti-inflammatory cytokines such as IL-10 and TGF-β play a protective role. These cytokines are involved in the resolution of inflammation and the promotion of tissue repair. Research indicates that IL-10 can inhibit the production of pro-inflammatory cytokines and reduce the activation of inflammatory cells, thereby mitigating the adverse effects of chronic inflammation on the cardiovascular system [20]. Additionally, TGF-β has been shown to promote collagen deposition and the stabilization of atherosclerotic plaques, which can prevent plaque rupture and subsequent acute coronary syndromes [21]. The balance between pro-inflammatory and anti-inflammatory cytokines is crucial in determining the overall inflammatory state of the vascular system. Disruptions in this balance may lead to an increased risk of coronary heart disease, highlighting the potential therapeutic implications of modulating cytokine levels in patients at risk for cardiovascular events [22].

#### The impact of cytokines on cardiomyocyte function

Cytokines significantly influence cardiomyocyte function, particularly in the context of ischemic heart disease. Pro-inflammatory cytokines such as TNF-α and IL-6 have been shown to induce cardiomyocyte apoptosis, impair contractility, and disrupt calcium handling, leading to heart failure [23]. The activation of the nuclear factor kappa-light-chain-enhancer of activated B cells (NF-κB) signaling pathway by these cytokines contributes to the inflammatory response and cellular dysfunction observed in ischemic conditions [24]. Conversely, anti-inflammatory cytokines like IL-10 exert protective effects on cardiomyocytes by promoting cell survival and reducing oxidative stress [25]. The dual role of cytokines—both detrimental and protective—highlights the complexity of their functions in cardiac physiology and pathology. Understanding these mechanisms is essential for developing targeted therapies that can modulate cytokine activity, potentially improving outcomes for patients with coronary heart disease [20].

### Cytokines and clinical research related to coronary heart disease

#### cytokine levels and risk assessment of coronary heart disease

Cytokines play a pivotal role in the pathogenesis of CHD by influencing inflammation, atherosclerosis, and myocardial ischemia. Research has shown that elevated levels of pro-inflammatory cytokines, such as IL-6 and TNF-α, are associated with an increased risk of developing CHD and its complications. For instance, a study found that patients with acute myocardial infarction (AMI) exhibited significantly higher IL-6 levels compared to those with stable angina, indicating a potential link between cytokine levels and the severity of coronary artery disease [22]. Furthermore, a Mendelian randomization study identified specific circulating inflammatory cytokines, including hepatocyte growth factor (HGF) and CCL20, as causal risk factors for coronary artery disease, highlighting the importance of cytokines in risk stratification and assessment [20]. In addition, previous studies on Mendelian randomization have shown a causal relationship between cytokines and the risk of coronary artery disease, indicating that genetic susceptibility to certain cytokine levels can lead to the development of specific types of cardiovascular diseases [26]. The association of cytokines with traditional risk factors, such as dyslipidemia and hypertension, reinforces their role in the multifactorial etiology of CHD. A prospective study by Ridker showed that after 8 years of follow-up, an increase in C-reactive protein levels increases the risk of cardiovascular disease [27]. Understanding these relationships can aid in developing targeted therapies and preventive strategies aimed at modulating cytokine levels to reduce cardiovascular risk. The perivascular fat attenuation index derived from cardiac computed tomography angiography can serve as an indirect marker of coronary artery inflammation and has demonstrated potential in predicting adverse cardiac events. Combining the assessment of coronary artery calcification score and perivascular fat attenuation index yields a comprehensive risk stratification approach that integrates the well-established calcification burden with novel inflammatory markers, thereby enhancing the prevention and management strategies for coronary artery disease [28].

#### Cytokines as prognostic biomarkers

Cytokines have emerged as promising prognostic biomarkers in coronary heart disease, providing insights into disease progression and outcomes. Elevated levels of inflammatory cytokines, such as IL-6 and C-reactive protein (CRP), have been correlated with adverse cardiovascular events, including heart failure and recurrent myocardial infarction. For example, a study demonstrated that high serum levels of IL-6 were associated with increased mortality in patients with ischemic heart disease [26]. Additionally, growth differentiation factor-15 (GDF-15) has been identified as a novel biomarker linked to oxidative stress and inflammation, showing prognostic value beyond traditional markers like natriuretic peptides [29]. The interplay between cytokines and other biomarkers, such as troponins and BNP, enhances the predictive accuracy for cardiovascular outcomes, enabling clinicians to stratify patients based on risk more effectively. The identification of cytokines as prognostic markers not only aids in risk assessment but also opens avenues for targeted therapies aimed at reducing inflammation and improving patient outcomes in CHD.

#### Clinical association between cytokines and coronary heart disease

Clinical trials investigating cytokine treatment interventions in coronary heart disease have yielded mixed results, reflecting the complexity of cytokine biology in cardiovascular disease. For instance, therapies targeting TNF-α and IL-1β have shown promise in reducing cardiovascular events in patients with chronic inflammatory conditions, such as rheumatoid arthritis, which is associated with increased cardiovascular risk [30]. The CANTOS trial demonstrated that targeting IL-1β with monoclonal antibodies significantly reduced cardiovascular events in patients with a history of myocardial infarction, underscoring the potential of anti-inflammatory strategies in managing coronary heart disease [30]. However, not all trials have been successful; some studies exploring the efficacy of remote ischemic preconditioning as a protective strategy against myocardial ischemia-reperfusion injury have reported disappointing outcomes, indicating that the application of cytokine modulation in clinical practice requires careful consideration of patient selection and timing of intervention [15]. Ongoing research aims to refine these approaches, focusing on the specific roles of various cytokines and their interactions within the inflammatory milieu of coronary heart disease, ultimately leading to more effective therapeutic strategies. There is relevant clinical study suggesting that moderate consumption of wine can significantly reduce cytokine levels in patients with coronary heart disease [31]. Through a case-control study, the participants were divided into three groups: control group, ethanol group, and wine group. The impact of alcohol consumption on coronary heart disease was evaluated by monitoring the cytokine levels in each group. Compared with the ethanol group, the wine group had lower secretion of TNF-α. The effect of ethanol intake on the secretion of cytokines by peripheral blood mononuclear cells in patients is weakened. This result suggests that appropriate use of wine can improve the progression of coronary heart disease by reducing the number of related cytokines.

### The significance of cytokines in the treatment of coronary heart disease

Cytokine-targeted therapy is gaining traction as a promising approach in the management of CHD, primarily due to the recognized role of inflammation in atherosclerosis. Cytokines, such as IL-6, TNF-α, and CRP, have been implicated as independent risk factors for cardiovascular diseases, including CHD [7]. Recent studies have highlighted the potential benefits of anti-inflammatory therapies targeting these cytokines, suggesting that reducing inflammation may slow the progression of atherosclerosis and improve clinical outcomes in patients with CHD [6]. For instance, the use of monoclonal antibodies against IL-1β has shown promise in reducing cardiovascular events in patients with a history of myocardial infarction [32]. Ziltivekimab is a narrow-spectrum fully human monoclonal antibody targeting the interleukin-6 (IL-6) ligand. Unlike other clinically available IL-6 signaling inhibitors, it was specifically developed for the management of atherosclerosis. RESCUE was a randomized, double-blind, phase II clinical trial evaluating ziltivekimab, the results of which demonstrated that ziltivekimab significantly reduces biomarkers of inflammation and thrombosis associated with atherosclerosis [33]. Findings from the randomized ZEUS clinical trial indicate that ziltivekimab reduces the incidence of cardiovascular events and may slow renal function decline. This represents a novel therapeutic strategy for patients with renal insufficiency and a history of myocardial infarction [34]. A meta-analysis on anti-inflammatory therapy for coronary heart disease summarized 1497 related studies, focusing on the comprehensive outcomes of all-cause mortality, recurrent myocardial infarction, and stroke, indicated that anti-inflammatory drugs seem to have a beneficial effect on reducing the risk of recurrent myocardial infarction in patients with coronary heart disease. The research results also demonstrated the protective effect of colchicine on the heart [35]. Tardif et al. carried out a randomized, double-blind clinical trial, and the results demonstrated that among patients with a recent myocardial infarction, colchicine at a dose of 0.5 mg daily led to a significantly lower risk of ischemic cardiovascular events than placebo [36]. In a randomized clinical trial conducted on patients diagnosed with chronic coronary disease, a notably reduced risk of cardiovascular events was observed in the cohort receiving a daily 0.5 mg dose of colchicine, in comparison to those administered a placebo [37]. In 2023, the U.S. Food and Drug Administration (FDA) approved low-dose colchicine (0.5 mg) for the reduction of cardiovascular risk. Subsequently, low-dose colchicine therapy for coronary heart disease has been incorporated into the 2023 American College of Cardiology (ACC)/American Heart Association (AHA) and 2024 European Society of Cardiology (ESC) clinical practice guidelines for chronic coronary syndromes [38, 39]. Therefore, colchicine is of great significance in reducing cardiovascular risks.

Furthermore, the anti-inflammatory effects of vitamin D in coronary heart disease have been validated. Clinical trials demonstrate that vitamin D therapy can reduce inflammatory responses and apoptosis following coronary artery bypass grafting by increasing levels of the anti-inflammatory cytokine IL-10 [40]. Similarly, α-lipoic acid has shown significant anti-inflammatory effects in patients with coronary heart disease complicated by type 2 diabetes, effectively lowering concentrations of C-reactive protein, IL-6, and TNF-α [41]. However, while the efficacy of these therapies is being explored, challenges remain, including the need for personalized treatment strategies and the potential for adverse effects from broad immunosuppression.

The development of novel cytokine inhibitors represents a frontier in the treatment of CHD, focusing on the modulation of specific inflammatory pathways. Researchers are investigating various agents that can selectively inhibit pro-inflammatory cytokines involved in atherosclerosis. For instance, monoclonal antibodies targeting IL-6 and TNF-α have been developed and are currently undergoing clinical trials to assess their effectiveness in reducing cardiovascular events [6]. Additionally, small-molecule inhibitors that disrupt cytokine signaling pathways are being explored. These include Janus kinase (JAK) inhibitors that can block the signaling of multiple cytokines simultaneously, thereby reducing inflammation and its associated cardiovascular risks [42]. The potential of these novel agents lies not only in their ability to mitigate inflammation but also in their capacity to improve endothelial function and stabilize atherosclerotic plaques [32]. As research progresses, the challenge will be to identify the most effective combinations of these therapies to maximize benefits while minimizing risks.

Combining cytokine-targeted therapies with other treatment modalities may enhance therapeutic outcomes for patients with coronary heart disease. The integration of anti-inflammatory cytokine therapies with traditional cardiovascular treatments, such as statins and antiplatelet agents, could provide a synergistic effect in managing atherosclerosis [43]. Statins, known for their lipid-lowering properties, also possess anti-inflammatory effects that may complement the action of cytokine inhibitors [32]. Furthermore, the exploration of lifestyle interventions, such as diet and exercise, alongside cytokine-targeted therapies, could optimize patient outcomes by addressing multiple risk factors simultaneously. Recent studies have indicated that lifestyle modifications can significantly reduce inflammatory markers and improve cardiovascular health, suggesting that a multifaceted approach may be the most effective strategy in managing CHD [44]. Future clinical trials will be essential to establish the efficacy and safety of such combination therapies, ultimately paving the way for more personalized treatment strategies in the management of coronary heart disease.

### Gaps and future research directions

Cytokines are pivotal in mediating immune responses and have multifaceted roles in various pathological conditions. Recent studies have highlighted the complexity of cytokine interactions, particularly in chronic inflammatory diseases such as atherosclerosis, where cytokines like IL-17 and IL-10 exhibit dual roles. For instance, IL-17, primarily secreted by Th17 cells, has been shown to promote atherosclerosis by enhancing inflammatory responses but may also stabilize atherosclerotic plaques under certain conditions, indicating a potential protective role [9]. Furthermore, the imbalance between regulatory T cells (Tregs) and Th17 cells is crucial in the progression of atherosclerosis, emphasizing the need for in-depth studies to unravel these dynamics. Investigating the multifunctional roles of cytokines could lead to novel therapeutic strategies that harness their beneficial effects while mitigating harmful outcomes. Future research should focus on elucidating the mechanisms by which cytokines exert these diverse effects, particularly in the context of immune regulation and chronic inflammation, to inform targeted therapies for diseases characterized by dysregulated cytokine signaling. The elevated levels of chemerin during acute myocardial infarction, combined with other inflammatory markers, highlight its potential as a diagnostic and prognostic biomarker. This dual effect highlights the potential of chemerin as an early detection and risk stratification target, enabling more personalized therapeutic interventions [45]. It has been demonstrated that S100A8/A9 holds potential for anti-inflammatory intervention. However, further research is required to elucidate the precise mechanism underlying the association between S100A8/A9 and coronary heart disease, as well as to validate therapeutic interventions targeting this pathway [46].

Technological advancements are revolutionizing the field of cytokine research, enabling more precise and comprehensive analyses of cytokine profiles and their functional implications. Techniques such as single-cell sequencing and multiplex cytokine assays allow researchers to dissect the complex interactions between various cytokines and immune cells at an unprecedented resolution [47]. Single cell sequencing technology, particularly single-cell (scRNA seq) or nuclear (snRNA seq) transcriptome analysis, has been proven to be an effective strategy for obtaining a global view of disease-related changes at unprecedented resolution [48]. By using single cell RNA sequencing, it was confirmed that Epsins in targeted pathological macrophages may provide therapeutic benefits for advanced atherosclerosis by reducing CD36 mediated lipid uptake and increasing ABCG1 mediated cholesterol outflow [49]. Moreover, the integration of artificial intelligence and machine learning in analyzing large datasets generated from high-throughput cytokine profiling can uncover novel therapeutic targets and biomarkers for disease progression. As research progresses, the development of multifunctional nanocarriers for targeted cytokine delivery also holds promise for enhancing therapeutic efficacy and reducing off-target effects in clinical settings [50]. Thus, the synergy between innovative technologies and cytokine research is expected to yield significant insights into immune regulation and therapeutic interventions. With the latest advances in artificial intelligence technology, machine learning methods that integrate and analyze various omics data are becoming increasingly valuable in the drug reuse of inflammatory diseases This review discusses the integration of advanced technologies, multi omics methods, and artificial intelligence (AI) in analyzing complex datasets to develop targeted therapies, as well as their potential to revolutionize the clinical aspects of malignant inflammation [51]. Previous study has analyzed the genetic profile changes associated with myocardial infarction [52]. So, whether there is a direct correlation between the relevant gene profile and cytokines can be used as a direction for future research.

Cytokines not only serve as biomarkers for disease diagnosis and prognosis but also play crucial roles in tailoring individualized treatment strategies. For instance, the Klotho/FGF23 axis inhibits cardiomyocyte apoptosis by regulating cytokine release through the ERK/MAPK pathway. Therefore, it can serve as a potential treatment for coronary heart disease [53]. Exosomes improve the healing of ischemic myocardial tissue by participating in intercellular communication processes, delivering useful substances such as cytokines from MSCs to recipient cells [54]. As research continues to elucidate the intricate roles of cytokines in health and disease, their integration into personalized medicine frameworks could lead to more effective and individualized therapeutic strategies. For example, the therapeutic effect of nano graphene oxide on atherosclerosis was evaluated by establishing an atherosclerosis model in vitro. From the perspective of precision medicine, this platform that uses patient derived vascular organoids can be further used for personalized drug screening in the future [55].

## Conclusion

Future research must focus on elucidating the dualistic nature of cytokines in CHD. This calls for innovative approaches to dissect their signaling pathways and interactions within the cardiovascular system. Emphasis should be placed on the development of novel therapeutic interventions that target cytokine signaling, with the aim of not only mitigating inflammation but also enhancing the reparative processes associated with cardiac injury.

In summary, the intricate role of cytokines in CHD underscores the necessity of a multifaceted research approach that incorporates diverse perspectives and findings. By advancing our understanding of cytokine biology and its implications in cardiovascular health, we can pave the way for innovative treatment strategies that not only improve the quality of life for patients but also enhance long-term prognoses in the face of this pervasive disease.

## Data Availability

No datasets were generated or analysed during the current study.

## Abbreviations

CHD: Coronary heart disease
TNF-α: Tumor necrosis factor-α
IL-1β: Interleukin-1 β
TCM: Traditional Chinese medicine
TGF-β: Transforming growth factor-β
NF-κB: Nuclear factor kappa-light-chain-enhancer of activated B cells
AMI: Acute myocardial infarction
HGF: Hepatocyte growth factor
CRP: C-reactive protein
GDF-15: Growth differentiation factor-15
JAK: Janus kinase
Tregs: Regulatory T cells
scRNA seq: Single-cell
snRNA seq: Nuclear
AI: Artificial intelligence
